# Roadside Air Quality Forecasting in Shanghai with a Novel Sequence-to-Sequence Model

**DOI:** 10.3390/ijerph17249471

**Published:** 2020-12-17

**Authors:** Dongsheng Wang, Hong-Wei Wang, Chao Li, Kai-Fa Lu, Zhong-Ren Peng, Juanhao Zhao, Qingyan Fu, Jun Pan

**Affiliations:** 1 Center for Intelligent Transportation Systems and Unmanned Aerial Systems Applications Research, State Key Laboratory of Ocean Engineering, School of Naval Architecture, Ocean and Civil Engineering, Shanghai Jiao Tong University, Shanghai 200240, China; dongshengwang_sjtu@163.com (D.W.); lichao_rusty@sjtu.edu.cn (C.L.); kaifalu@sjtu.edu.cn (K.-F.L.); 2 International Center for Adaptation Planning and Design, College of Design, Construction and Planning, University of Florida, P.O. Box 115706, Gainesville, FL 32611-5706, USA; 3 Department of Computer Science, Viterbi School of Engineering, University of Southern California, Los Angeles, CA 90089, USA; juanhaoz@usc.edu; 4 Shanghai Environmental Monitoring Center, Shanghai 200235, China; qingyanf@sheemc.cn (Q.F.); panj@sheemc.cn (J.P.)

**Keywords:** roadside air quality forecasting, deep learning, sequence to sequence, short-term prediction, fine particulate matter, carbon monoxide

## Abstract

The establishment of an effective roadside air quality forecasting model provides important information for proper traffic management to mitigate severe pollution, and for alerting resident’s outdoor plans to minimize exposure. Current deterministic models rely on numerical simulation and the tuning of parameters, and empirical models present powerful learning ability but have not fully considered the temporal periodicity of air pollutants. In order to take the periodicity of pollutants into empirical air quality forecasting models, this study evaluates the temporal variations of air pollutants and develops a novel sequence to sequence model with weekly periodicity to forecast air quality. Two-year observation data from Shanghai roadside air quality monitoring stations are employed to support analyzing and modeling. The results conclude that the fine particulate matter (PM_2.5_) and carbon monoxide (CO) concentrations show obvious daily and weekly variations, and the temporal patterns are nearly consistent with the periodicity of traffic flow in Shanghai. Compared with PM_2.5_, the CO concentrations are more affected by traffic variation. The proposed model outperforms the baseline model in terms of accuracy, and presents a higher linear consistency in PM_2.5_ prediction and lower errors in CO prediction. This study could assist environmental researchers to further improve the technologies for urban air quality forecasting, and serve as tools for supporting policymakers to implement related traffic management and emission control policies.

## 1. Introduction

Traffic emissions have been one of the major contributors to urban air pollution in many cities around the world [1,2], and can deteriorate ambient air quality on a wide range of spatial scales. Epidemiological studies indicate that long-term exposure to traffic-related air pollution could harm human health [3], lead to respiratory and cardiovascular diseases, and even increase mortality [4,5]. Even short-term exposure to ambient particulate air pollution could greatly increase the risk of myocardial infarction [6]. In addition, the fine particulate matter (PM_2.5_) can also notably reduce visibility [7] and thus affects road capacity and traffic safety. Hence, it is crucial to build an efficient and accurate air quality forecasting system that could help the department of transportation to broadcast warning information to travelers, and provide guides to residents to make better plans for outdoor activities and avoid health-threatening travels. Therefore, in order to protect public health and improve traffic safety, it is necessary to develop efficient prediction models to forecast air quality.

There are two main types of models in forecasting air quality: deterministic models based on atmospheric physics and chemical mechanisms, and empirical models based on statistics and machine learning. Deterministic models, such as weather research and forecasting model coupled with chemistry (WRF-Chem) [8,9] and community multi-scale air quality model (CMAQ) [10], could help explain the formation, development, and transport of air pollutants. However, the performance of these models could be affected by the uncertainty of emissions and chemical reactions, which further weakens the ability to model spatial and temporal resolution [11]. The input parameters of the deterministic models (e.g., emission inventory) commonly contain very limited information of pollution sources, lack spatial and temporal dependencies for some air pollutants [12], and also present strong difficulty when being updated in time due to the high cost. These disadvantages could notably affect the prediction performance of the deterministic models.

Empirical models can provide more accurate predictions with relatively few parameters based on the theories of statistics and machine learning. The autoregressive integrated moving average (ARIMA) model has been widely used for time series analysis. A study of PM_2.5_ forecasting in Beijing demonstrates that the ARIMA model achieves better results in the next 1-h prediction with a lower error [13]. However, when the time lag expands, the prediction results become worse. Support vector regression (SVR) has also been employed for air quality prediction, and evolutionary algorithms, as an import tool for parameter optimization [14,15], are used to improve the empirical models. For example, when the quantum-behaved particle swarm optimization (QPSO) algorithm is used to determine the input parameters for the SVR model, the hybrid QPSO-SVR model could present better prediction performance in both computational time and model accuracy [16]. Moreover, the support vector regression model considering the space-time factors outperforms the traditional SVR model in accuracy [11].

Recently, deep learning has exhibited the potentials of enhancing the methodology of resilience assessment, and demonstrated strong performances in air pollution forecasting, due to its flexible model structure and powerful learning ability [17,18,19,20]. Furthermore, with the improvement of training algorithms and computing performances, the artificial neural network (ANN) has been made possible to be trained as more dense and multi-layered neural networks, such as the deep neural network (DNN). The DNN can be widely applied for a variety of tasks, especially for modeling complex nonlinear relationships, such as stacked autoencoder (SAE) [21], recurrent neural network (RNN) [22], gated recurrent unit (GRU) [23], and long short-term memory (LSTM) neural network [24]. A study on the prediction of the hourly PM_2.5_ concentrations in Beijing shows that the prediction accuracy of LSTM is significantly better than that of ARMA and SVR models [25]. A long short-term memory-fully connected (LSTM-FC) neural network also performs well in predicting the PM_2.5_ concentrations in Beijing [26]. An attention-based air quality predictor (AAQP) model, known as a variant of the Seq2Seq model, shows that the prediction accuracy of AAQP in Beijing outperforms that of the traditional LSTM and the basic Seq2Seq model [27]. Also, the LSTM and Seq2seq models demonstrate worse prediction performances when the time lags become longer. Although the deep learning models mentioned above can effectively capture the spatiotemporal dependencies in air quality predictions, the characteristics of the time series itself (such as periodicity) are still insufficiently incorporated into these models. Actually, the roadside air quality is directly affected by traffic-related pollutants, and the temporal variation is strongly consistent with the diurnal pattern of traffic flow. Specifically, the diurnal variation of traffic-related particles suggests two peaks, which is strongly associated with intensive emissions during two traffic rush hours [28]. If the periodicity consistent with traffic flow patterns is fully considered in modeling, the deep learning models could be more suitable for roadside air quality forecasting with a higher accuracy. Therefore, it is necessary to develop an advanced deep learning model including periodic features of the time series.

To address this issue, we propose a novel sequence to sequence (Seq2Seq) model with weekly periodicity to predict the traffic-related PM_2.5_ and CO concentrations. In this study, we analyze the hourly variations of the PM_2.5_ and CO concentration data from two roadside air quality monitoring stations in Shanghai. Then, the Seq2Seq model with weekly periodicity is developed by incorporating the diurnal variation patterns and taking full account of the periodic characteristics of pollutants. Finally, the 24-h prediction results are compared with the baseline models. Among all the air pollutants, fine particulate matter (PM_2.5_) and carbon monoxide (CO) are selected in this paper mainly for the two following reasons: Firstly, PM_2.5_ is one of the most important air pollutants in megacities [29], and CO is a stable gaseous pollutant and mainly results from traffic emissions [30]. Secondly, PM_2.5_ and CO represent two different kinds of air pollutants, namely particles and gaseous pollutants. Therefore, the characterization of the two air pollutants could represent different temporal patterns of traffic emissions and also assist in evaluating the prediction performance of the proposed model responding to varying pollutants [31].

## 2. Data and Methods

### 2.1. Study Area and Data Description

**Figure 1:**
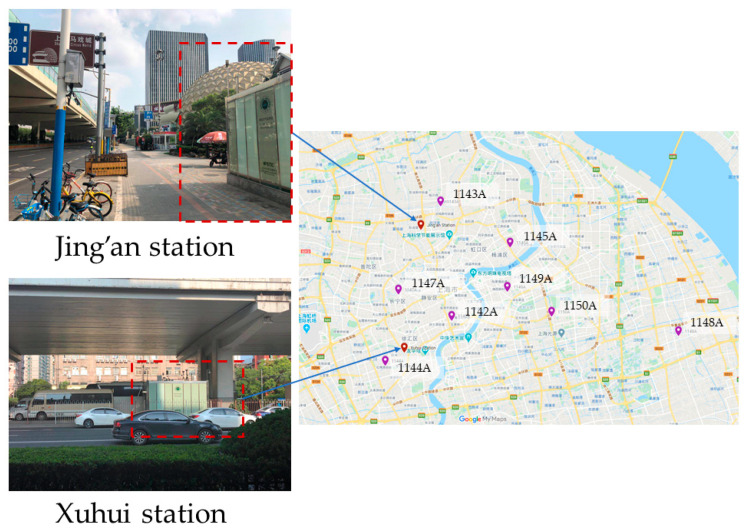
The spatial locations of roadside and urban air quality monitoring stations.

Shanghai, a megacity located in the Yangtze River Delta, is one of the most economically developed regions in China. Although industrial emissions are still the major local contributors to the total PM_2.5_ concentrations in Shanghai, vehicle emissions contribute more to particulate pollution in urban areas [32]. Therefore, it is reasonable to select Shanghai as the study area to analyze the temporal variations of air pollutants, especially for local traffic-related air pollution. The roadside air quality is more affected by traffic emissions and has been generally considered as serious pollution hotspots under common concern [33]. In this paper, we choose two roadside air quality monitoring stations for case studies. The two monitoring stations are affiliated with the Shanghai Environmental Monitoring Center (SEMC) and adjacent to urban arterial roads. The design intent of the two monitoring stations mainly focuses on analyzing the impacts of road traffic emission sources on ambient air quality. The locations of the roadside air quality monitoring stations are shown as red markers in Figure 1. The hourly pollutant data from 8 extra monitoring stations over urban areas of Shanghai are also used to verify the external validity of the air quality forecasting model subsequently proposed, and their locations are shown as purple markers in Figure 1.

The hourly data of six air pollutants from 1 March 2015 to 28 February 2017 are used in this study, such as particulate matter less than 2.5 μm (PM_2.5_) and less than 10 μm (PM_10_), carbon monoxide (CO), nitrogen dioxide (NO_2_), ozone (O_3_), and sulfur dioxide (SO_2_). The hourly mass concentrations of air pollutants are measured by automated monitoring instruments in the air quality monitoring stations. The daily quality control and quality assurance (QC/QA) are implemented by the professional staff of SEMC [34], according to the Technical Specifications for Operations and Quality Control of the Ambient Air Quality Automated Monitoring System for Particulate Matter (PM_10_ and PM_2.5_) and the Technical Specifications for Operations and Quality Control of the Ambient Air Quality Automated Monitoring System for SO_2_, NO_2_, O_3_, and CO, which are issued by the Ministry of Ecology and Environment of the People’s Republic of China.

Among the six air pollutants, PM_2.5_ (Shanghai’s major pollutant) and CO (closely related to traffic emissions) are chosen as the main research objects. Other pollutants are input as auxiliary parameters in the air quality forecasting model. The meteorological data are generated by the Weather Research and Forecasting (WRF) model [8] in the Yangtze River Delta region with a grid resolution of 5 × 5 km. The grid data closest to each air quality monitoring station are selected to geographically match the meteorological and pollutant data, and then build the dataset. The valid meteorological data (e.g., temperature, humidity, air pressure, wind, and precipitation) are also adopted to further strengthen the prediction performance of the proposed model.

### 2.2. Autocorrelation Analysis

Autocorrelation, also known as serial correlation, refers to the calculation of the correlation between one time series and the previous ones. When the time series contain periodic components, the maximum value of the autocorrelation function will be an indicator of periodicity. The autocorrelation function is defined as follows [35]:

(1) $$r_{k}=\;\frac{c_{k}}{c_{0}}$$

where $c_{k}=\frac{1}{T}\overset{T-k}{\underset{t=1}{\sum }}\left(y_{t}-\bar{y}\right)\left(y_{t+k}-\bar{y}\right)$, k is the time lag, and $y_{i}$ represents the *i*-th value of the time series.

### 2.3. Long Short-Term Memory (LSTM) Networks

Air pollution data from ground-based monitoring stations are generally recorded as the time-series data. Thus, the recurrent neural networks (RNNs) are better suited to air quality forecasting than the feed-forward neural networks (FNNs) applied in previous studies [36]. Vanilla recurrent neural networks suffer from the vanishing gradient and exploding gradient problems during long sequence training, which could be relieved by long short-term memory (LSTM) networks due to the gated mechanism [37].

**Figure 2:**
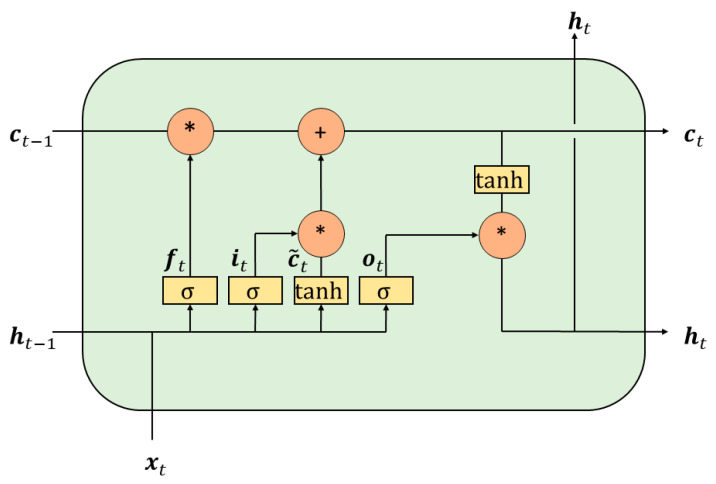
The structure of LSTM networks.

Figure 2 shows the structure of LSTM networks. In each LSTM cell, there is an input gate ($i_{t}$), a forget gate ($f_{t}$), and an output gate ($o_{t}$). The input gate, the forget gate, and the output gate control new information adding, previous information preserving, and prediction outputting, respectively. The equations for the hidden state transferring are shown in Equations (2)–(7):

(2) $$i_{t}\;=\;\sigma \left(W_{i}x_{t}+U_{i}h_{t-1}+b_{i}\right)$$

(3) $$f_{t}\;=\;\sigma \left(W_{f}x_{t}+U_{f}h_{t-1}+b_{f}\right)$$

(4) $$o_{t}\;=\;\sigma \left(W_{o}x_{t}+U_{o}h_{t-1}+b_{o}\right)$$

(5) $${\tilde{c}}_{t}\;=\;tanh\left(W_{c}x_{t}+U_{c}h_{t-1}+b_{c}\right)$$

(6) $$c_{t}\;=\;f_{t}*c_{t-1}+i_{t}*{\tilde{c}}_{t}$$

(7) $$h_{t}\;=\;o_{t}*tanhc_{t}$$

where $x_{t}$ is the input to the cell at time t, $c_{t}$ is the cell state, and $h_{t}$ is the hidden state. σ refers to the sigmoid function. $W_{i}$, $W_{f}$, $W_{o}$, $W_{c}$, $U_{i}$, $U_{f}$, $U_{c},$ and $U_{o}$ represent different weights, respectively, and $b_{i}$, $b_{f}$, $b_{o}$, $b_{c}$ separately represent bias terms. The symbol tanh denotes hyperbolic tangent function:

(8) $$tanh\left(x\right)=\frac{e^{x}-e^{-x}}{e^{x}+e^{-x}}$$

and the operator ∗ in Equations (6) and (7) refers to the Hadamard product.

### 2.4. Sequence to Sequence (Seq2Seq) Model

**Figure 3:**
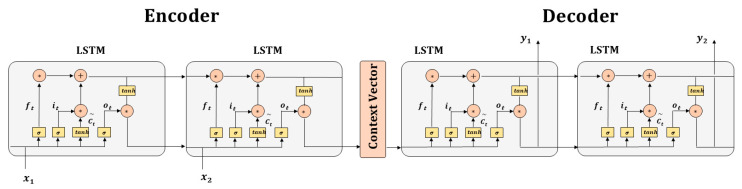
The architecture of the Seq2Seq model.

The sequence-to-sequence (Seq2Seq) model was developed by Sutskever et al. for machine translation [38], and Cho et al. subsequently refined the model [39]. The Seq2Seq model is an Encoder-Decoder structure with sequential inputs and outputs, and generally recurrent neural networks (e.g., vanilla RNN, LSTM, or GRU, etc.) are employed to build the encoder and decoder. The architecture of the Seq2Seq model is shown in Figure 3. Specifically, input sequences are read and encoded to a context vector by the encoder RNN, and then output sequences are generated from the vector by the decoder RNN [38]. Here, $h_{t}\in R^{m}$ denotes the hidden state at past timestep t in the encoder, where m refers to the size of the context vector. Similarly, $s_{t^{\prime }}\in R^{n}$ and n represent the hidden state at a future timestep $t^{\prime }$ in the decoder. The context vector c in the decoder is a weighted sum of all hidden states with T timesteps in the encoder, as shown:

(9) $$h_{t}=f\left(x_{t},h_{t-1}\right)$$

(10) $$c=g(\{h_{1},\;\ldots ,h_{T}\})$$

where f and g both refer to nonlinear functions. The decoder RNN is trained to predict the output $y_{t^{\prime }}$ at each future timestep $t^{\prime }$ by considering context vector c and past output {$y_{1},\;\ldots ,\;y_{t^{\prime }-1}$}. In RNN units, previous long-term output {$y_{1},\;\ldots ,\;y_{t^{\prime }-2}$} can be stored and updated in the hidden state $s_{t^{\prime }-1}$ of the decoder RNN, and thus the output sequence at $t^{\prime }$ time is presented as:

(11) $$y_{t^{\prime }}=\;p\left(y_{t^{\prime }-1},s_{t^{\prime }},c\right)$$

(12) $$s_{t^{\prime }}=\;q\left(y_{t^{\prime }-1},s_{t^{\prime }-1},c\right)$$

where p and q both refer to nonlinear functions.

**Figure 4:**
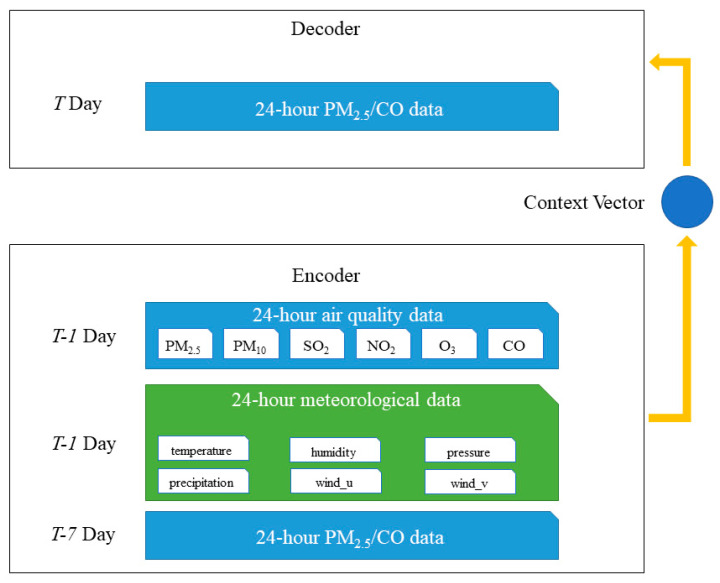
The framework of the Seq2Seq model with weekly periodicity.

In this study, the structure of LSTM networks is chosen for building the encoder and the decoder, and the framework of the proposed model is shown in Figure 4. The input of the LSTM encoder is a sequence with 24 time-steps, namely a 24-h time series. For a better understanding of the input sequence with a variety of parameters, the input sequence can be considered as an integration of three different sequences: the air quality data for day *T*, the meteorological data for day *T-1*, the PM_2.5_ or CO data for day *T-7*. The air quality data and meteorological data for day *T-1* are chosen with reference to the selections of other input parameters for data-driven air quality forecasting models in previous studies [11,26]. The additional air quality data for day *T-7* are used to reflect the weekly periodicity in our model. The LSTM networks, as the decoder, are also used to generate the output from the hidden state (a context vector). The output is a sequence of 24-h air quality (PM_2.5_ or CO) and presents the same length as the input sequence.

## 3. Results and Discussion

### 3.1. Diurnal Variation

**Figure 5:**
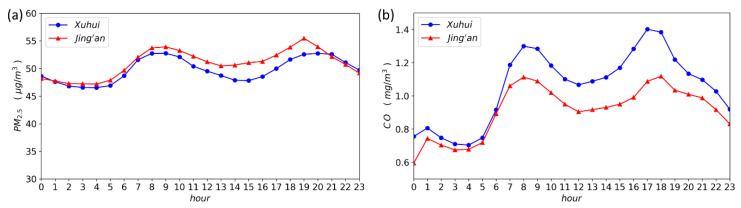
The diurnal variations of air quality data in the two monitoring stations. (**a**) PM_2.5_ (**b**) CO.

Before air quality forecasting, we first analyze the temporal characteristics of the PM_2.5_ and CO concentrations. As illustrated in Figure 5, the diurnal variation patterns of the hourly-average PM_2.5_ concentrations exhibit two distinct peaks and valleys, and the peaks and valleys of the two air quality monitoring stations show slight differences. This pattern can also be found for the daily variation of the CO concentrations.

The diurnal trend revealed in this study is also consistent with that reported in Shanghai by related research [40]. For PM_2.5_, the two peak concentrations separately appear at 8–9 a.m. and 7–8 p.m. For CO, the two peaks occur at 8 a.m. and 5–6 p.m., respectively. Coincidentally, there are two traffic rush hours, namely at 8 a.m. and 6 p.m., in terms of the number of trips in Shanghai [41]. It can be found that the CO peaks observed in Shanghai are close to the peaks of traffic volume, while the PM_2.5_ peaks are delayed by about one hour. This finding indicates that CO mainly results from traffic emissions, and thus exhibits similar diurnal patterns to the changes in traffic volume in one day. For the time lag of the PM_2.5_ peaks, one possible explanation is that vehicle-emitted primary particles are mainly composed of ultrafine particles [42], which are in Aitken nuclei mode, and it takes time to grow into the accumulated mode by coagulation and condensation.

**Figure 6:**
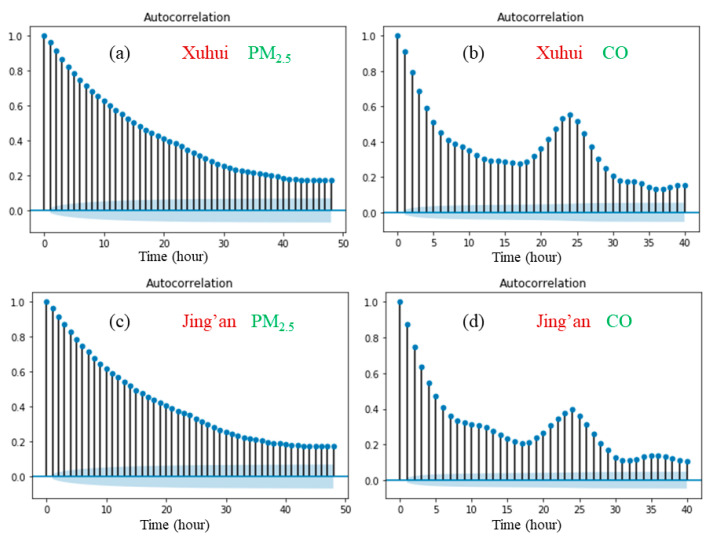
Autocorrelation function (with 95% confidence intervals) of hourly air quality data in the two monitoring stations. (**a**) Xuhui, PM_2.5_ (**b**) Xuhui, CO (**c**) Jing’an, PM_2.5_ (**d**) Jing’an, CO.

Next, an autocorrelation analysis based on Equation (1) is performed to verify whether the temporal variations of the PM_2.5_ and CO concentrations exhibit the daily periodicity, and the results are shown in Figure 6. It can be easily recognized from Figure 6 that the CO concentrations exhibit an apparent 24-h periodicity at the two monitoring stations, while the PM_2.5_ concentrations hardly show any daily periodicity.

As shown in Figure 6, the autocorrelation coefficient of CO at Xuhui station is higher than that at Jing’an station, which indicates a more significant daily periodicity of CO at Xuhui station. Considering that the CO exhibits more similar daily periodicity to the traffic flow, we consider that the CO concentrations measured at Xuhui station present a more significant daily periodicity. This can also be explained by the fact that the Xuhui station is located on the separation zone between the two-way lanes, and is closer to the traffic emission sources in both directions. However, Jing’an station is located on the roadside, relatively far from the on-road traffic flow. Therefore, the air quality data from Xuhui station are more affected by the on-road traffic flow patterns than those from Jing’an station.

The phenomenon of a lack of periodicity of PM_2.5_ mainly lies in the generation of PM_2.5_ being so complicated that the periodicity is not obvious. On the other hand, the results suggest that there are no linear relationships between the time series of the PM_2.5_ concentrations and one lagged version of the time series.

### 3.2. Weekly Variation

The average PM_2.5_ concentrations exhibit a significant weekly variation, with a fluctuation magnitude of about 10 μg/m^3^ between the peak and the valley. Besides, the weekly variations of the PM_2.5_ concentrations show two peaks and one valley, which successively appear on Friday, on Sunday, and on Tuesday. Furthermore, the CO concentrations also show a similar weekly trend. Unlike other cities, for example, the PM_2.5_ concentrations in Beijing on weekdays are higher than the weekend, while the PM_2.5_ concentrations in Shanghai hardly show the “weekend effect”, which could be further confirmed by previous studies [43].

**Figure 7:**
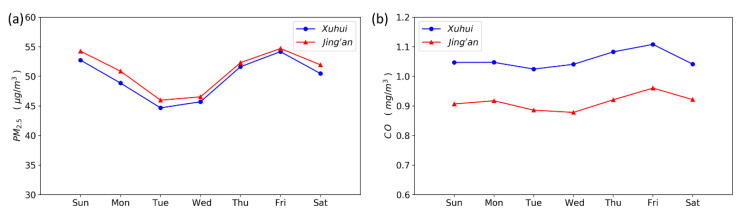
Weekly variation of the average PM_2.5_ concentration in the two monitoring stations. (**a**) PM_2.5_ (**b**) CO.

**Figure 8:**
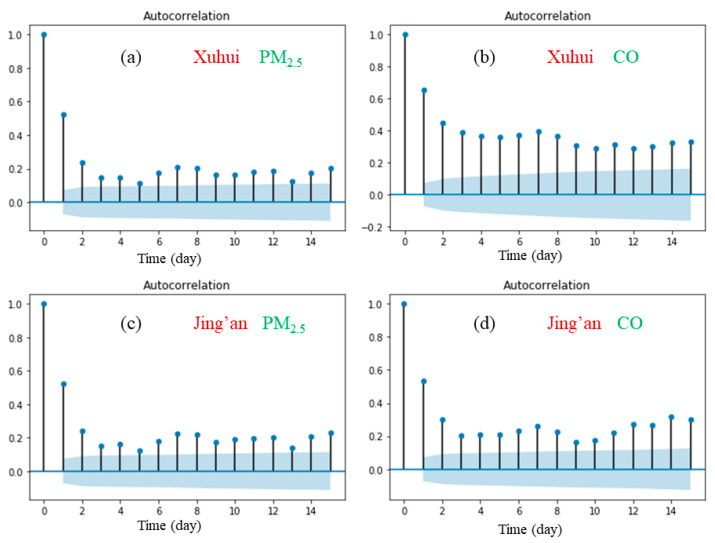
Autocorrelation function (with 95% confidence intervals) of daily air quality data in the two monitoring stations. (**a**) Xuhui, PM_2.5_ (**b**) Xuhui, CO (**c**) Jing’an, PM_2.5_ (**d**) Jing’an, CO.

We also perform the autocorrelation analysis to investigate the periodic variations in the daily average concentrations of PM_2.5_ and CO, and the results are shown in Figure 7. It can be found that the daily average concentrations of PM_2.5_ and CO both exhibit a significant weekly periodicity. In addition, Figure 8 suggests that there is a linear relationship between the time series and its lagged version of seven days. This result also implies that this linear relationship could be integrated into our proposed model to improve the forecasting accuracy.

In Figure 5 and Figure 7, the daily and weekly variations of pollutant concentrations measured at the two monitoring stations demonstrate some differences in terms of the pollution characteristics between the two monitoring stations. The CO concentrations of the Xuhui station are significantly higher than those of the Jing’an station, especially during the traffic rush hours. The PM_2.5_ concentrations are nearly equivalent at the two monitoring stations, and the PM_2.5_ concentrations of the Jing’an station are slightly higher than those of the Xuhui station. Besides, the higher CO concentrations at the Xuhui station could be explained by the fact that the station is located closer to the traffic emission sources, while the Xuhui station is located below the elevated road, which prevents the upward dispersion of traffic-emitted gaseous pollutants at the ground level. The difference in the PM_2.5_ concentrations between the two monitoring stations is negligible, indicating that the PM_2.5_ concentrations are affected by both local traffic emission sources and regional air pollution.

### 3.3. Forecasting Model Results

To evaluate the prediction performance of the Seq2Seq model with weekly periodicity, several machine learning models are selected as the comparison models: Seq2Seq model without weekly periodicity, Bidirectional LSTM [44], and LSTM [37]. All the models are implemented in Python with Keras, pandas, NumPy and scikit-learn in a Linux system. We select three statistical indices to evaluate the prediction performance of the models: root mean square error (RMSE), normalized mean square error (NMSE) and Pearson correlation coefficient (r), which are defined as:

(13) $$\mathrm{RMSE}=\;\sqrt{\frac{1}{n}\sum_{i=1}^{n}{\left(O_{i}-P_{i}\right)}^{2}}$$

(14) $$NMSE=\frac{\bar{{\left(P_{i}-O_{i}\right)}^{2}}}{\bar{P}\cdot \bar{O}}$$

(15) $$r=\;\frac{\sum_{i=1}^{n}\left(O_{i}-{\bar{O}}_{i}\right)\left(P_{i}-{\bar{P}}_{i}\right)}{\sqrt{\sum_{i=1}^{n}{\left(O_{i}-{\bar{O}}_{i}\right)}^{2}}\sqrt{\sum_{i=1}^{n}{\left(P_{i}-{\bar{P}}_{i}\right)}^{2}}}$$

where P and O, respectively, refer to the prediction value and the observed value, and ${\bar{P}}_{i}$ and ${\bar{O}}_{i}$ separately denote the mean of the prediction value and the observed value.

The model results demonstrate that the Seq2Seq model with weekly periodicity shows better prediction performances, as shown in Table 1, with lower errors (RMSE and NMSE) and a higher correlation coefficient (r). In addition, all the machine learning models present lower errors in CO forecasting, compared with PM_2.5_ forecasting. However, in terms of the correlation coefficient, these models perform better in PM_2.5_ forecasting than CO forecasting.

In Table 1, the prediction accuracy of both the PM_2.5_ and CO concentrations at the Xuhui station is higher than that at the Jing’an station. From the perspective of NMSE, the Xuhui station shows better improvements in the prediction accuracy of CO than the Jing’an station, which could be partly explained by the periodicity of the air quality data at the Xuhui station discussed in Section 3.1 and Section 3.2 To be specific, Figure 6 indicates that the 24-h periodicity (autocorrelation coefficient) at the Xuhui station is higher than that at the Jing’an station. In addition, Figure 8 also shows the seven-day autocorrelation coefficient of CO at the Xuhui station is significantly higher than that of the Jing’an station. The results demonstrate that the air quality data of the Xuhui station exhibit more obvious periodicity. Furthermore, the obvious periodicity mainly lies in that the Xuhui station is located in the separation zone between two-way roads, just under the elevated expressway, and could be more affected by traffic emissions. The periodicity result demonstrates that the air quality data at the Xuhui station exhibit a stronger regularity and predictability, and thus achieve a higher model prediction accuracy.

The model performances of Seq2Seq with weekly periodicity under different input parameters are presented in Table 2. It can be found that meteorological factors play an important role in improving the prediction accuracy of the proposed model. Moreover, the other four pollutants also contribute to the improvement of the model performance.

**Figure 9:**
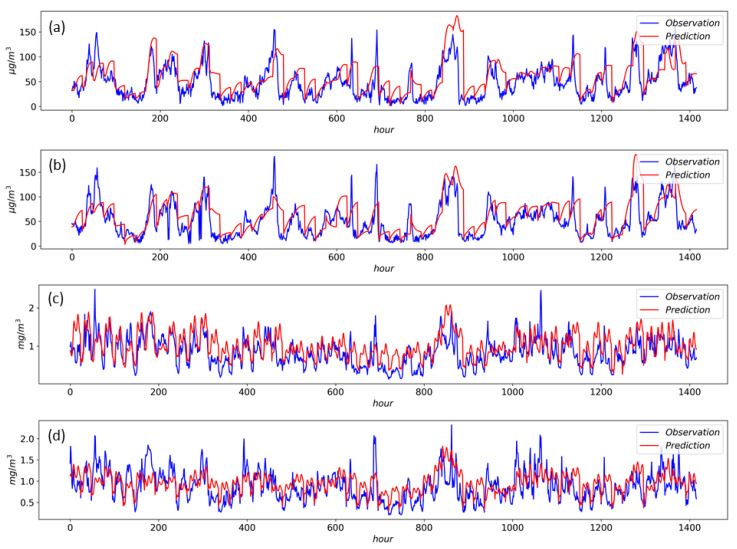
Forecasting results from 1 January to 28 February 2017. (**a**) Xuhui, PM_2.5_ (**b**) Jing’an, PM_2.5_ (**c**) Xuhui, CO (**d**) Jing’an CO.

For an intuitive understanding of the model results, we select the measured and forecast results from 1 January to 28 February 2017 and then draw a comparison of the predicted and observed values, as shown in Figure 9. The predicted values of the CO concentrations present a similar periodicity with the observed data, and the temporal variation shows relative consistency between the prediction and observation values. The predicted values of the PM_2.5_ concentrations show a relatively smooth time-varying trend, although there are lots of mutations in the time-series sequences of the observed data. However, the forecast results under heavily polluted conditions (e.g., peak values) need to be further improved when using the proposed model in PM_2.5_ forecasting.

### 3.4. Urban Monitoring Station Results

**Figure 10:**
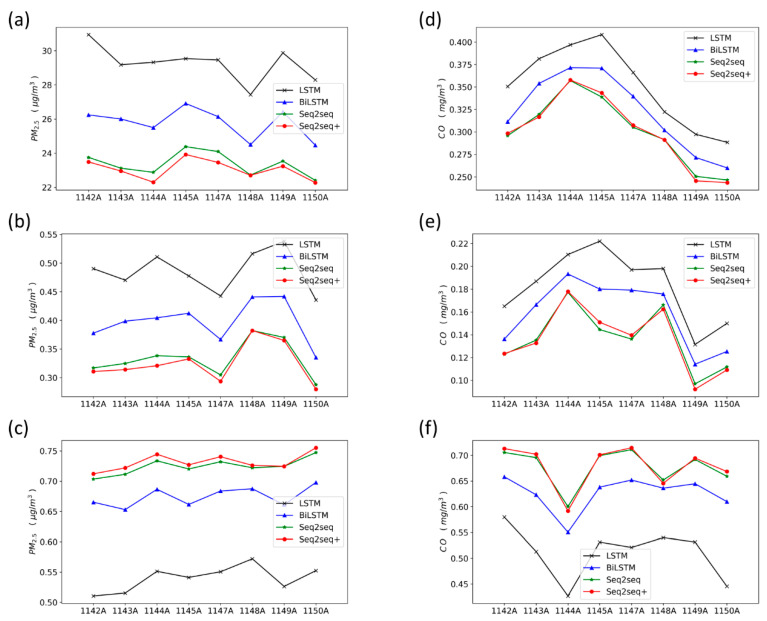
Comparison of the forecasting model results for 8 urban air quality monitoring stations. (**a**) PM_2.5_, RMSE (**b**) PM_2.5_, NMSE (**c**) PM_2.5_, r (**d**) CO, RMSE (**e**) CO, NMSE (**f**) CO, r.

To verify whether the proposed model can be applied to other general circumstances, we select the eight extra urban monitoring stations in Shanghai to analyze the external validity of the proposed model. Detailed description of these stations is shown in Table 3. The prediction results of the four models at the eight urban monitoring stations are shown in Figure 10. In Figure 10, the Seq2Seq model with periodicity generally performs better in PM_2.5_ forecasting than other models at most sites, and also presents lower errors and a higher correlation coefficient. However, the prediction performance of the proposed model in CO forecasting is similar to that of the traditional Seq2seq model.

As shown in Figure 10, the weekly periodicity significantly improves the model accuracy in PM_2.5_ forecasting, but shows little improvements in CO forecasting. One possible explanation is that the temporal variation of the CO concentrations exhibits the weekly periodicity and obvious daily periodicity. Although the daily periodicity has also been considered by revising the format of the input data in the baseline models, the extra weekly periodicity of CO could only provide minor improvements. Overall, according to the model performances at the state control points, the Seq2Seq model with weekly periodicity could be widely used for air quality forecasting at other monitoring stations, especially for PM_2.5_ forecasting.

## 4. Conclusions

In order to address issues of neglecting temporal periodicity in previous studies, a novel Seq2Seq model with weekly periodicity is developed to forecast roadside air quality. We analyze the daily and weekly variation patterns of roadside air quality data to clearly understand the underpinnings of the proposed model. To consider the characteristics of weekly periodicity, we build the Seq2Seq model with weekly periodicity by adding an auxiliary air quality sequence from a week ago into the present model. Six kinds of air pollutants from roadside air quality monitoring stations in Shanghai are employed in the model, as well as six meteorological parameters from WRF model. To further verify the external validity of the model, eight auxiliary urban air quality monitoring stations are also used in this study.

There are several general findings concluded in this research:

(1) The daily trend of CO and PM_2.5_ is consistent with the trend of daily traffic volume in Shanghai, and the PM_2.5_ suggests a strong hysteresis (roughly one hour). Morning and evening traffic rush hours are also high pollution-level periods.
(2) The concentrations of air pollutants are quite different between the two roadside air quality monitoring stations Xuhui and Jing’an, and the latter is lightly affected by traffic emissions. The CO concentrations at the Xuhui station are higher, while the PM_2.5_ concentrations at the two stations are similar. Given that the Xuhui station is closer to the traffic emission sources than the Jing’an station, the results indicate that the CO concentrations at the two stations are mainly affected by local traffic sources, and the pollution sources of PM_2.5_ are not only limited to local traffic emissions.
(3) This model improves the forecasting accuracy of the roadside air quality, compared with the traditional Seq2Seq model and other baseline machine learning models. The model accuracy is higher at the Xuhui station where the periodic characteristics of air pollutants are more noticeable. This result suggests that the daily periodicity caused by traffic should not be overlooked in modeling and forecasting air quality in roadside areas. Therefore, the weekly periodicity should be fully considered in air quality forecasting.
(4) The proposed Seq2Seq model with weekly periodicity was also suitable for the eight urban monitoring stations in Shanghai. In contrast, the weekly periodicity demonstrates a more pronounced impact on PM_2.5_ forecasting. For CO forecasts, the weekly periodicity-based model is not necessarily appropriate for all monitoring stations.

The major contributions of this research are listed as below:

(1) The temporal patterns of traffic and air quality are fully evaluated and further summarized based on the two-year air quality monitoring data in megacities;
(2) Weekly periodicity is taken into account in the deep learning-based air quality forecasting model, which strongly improves prediction accuracy;
(3) The proposed Seq2Seq model with weekly periodicity is also applicable to urban air quality prediction (not only for traffic-related roadside air quality), and thus can be used by public authorities to make timely management adjustments to protect public health based on air quality predictions.

In terms of implication, the proposed Seq2Seq model with weekly periodicity shows that the weekly periodicity significantly improves the predictability of the deep learning model focusing on air quality forecasting. One limitation of this research is that only the temporal patterns of data from monitoring stations are considered, but the spatial dependencies between stations are not integrated into the model. Future studies are expected to incorporate more spatiotemporal features and further consider the topological correlations among air quality monitoring stations when developing deep learning models.

## Figures and Tables

**Table 1:** Comparison of the forecasting model results.

Station	Model	PM_2.5_			CO		
		RMSE	NMSE	r	RMSE	NMSE	r
Xuhui	Seq2Seq with weekly periodicity	21.51	0.281	0.735	0.327	0.124	0.681
	Seq2Seq	27.13	0.335	0.692	0.349	0.132	0.676
	Bidirectional LSTM	27.66	0.360	0.641	0.355	0.146	0.577
	LSTM	30.49	0.432	0.540	0.364	0.147	0.581
Jing’an	Seq2Seq with weekly periodicity	23.14	0.278	0.716	0.348	0.160	0.590
	Seq2Seq	25.73	0.312	0.683	0.360	0.173	0.587
	Bidirectional LSTM	24.28	0.345	0.667	0.362	0.174	0.565
	LSTM	33.13	0.503	0.482	0.370	0.202	0.475

**Table 2:** The model performances of Seq2Seq with weekly periodicity under different input parameters.

Station	Model Input Parameters	PM_2.5_			CO		
		RMSE	NMSE	r	RMSE	NMSE	r
Xuhui	PM_2.5_ & CO	25.92	0.377	0.695	0.353	0.134	0.654
	PM_2.5_ & CO + POL ^1^ (6 pollutants)	24.85	0.321	0.647	0.322	0.121	0.661
	PM_2.5_ & CO + MEO ^2^	21.93	0.293	0.720	0.305	0.108	0.704
	PM_2.5_ & CO + POL + MEO (all)	21.51	0.281	0.735	0.327	0.124	0.681
Jing’an	PM_2.5_ & CO	25.45	0.365	0.661	0.352	0.165	0.577
	PM_2.5_ & CO + POL (6 pollutants)	24.59	0.322	0.672	0.355	0.161	0.606
	PM_2.5_ & CO + MEO	23.28	0.334	0.713	0.353	0.157	0.616
	PM_2.5_ & CO + POL + MEO (all)	23.14	0.278	0.716	0.348	0.160	0.590

^1^ POL: PM_10_, SO_2_, NO_2_, and O_3_. ^2^ MEO: temperature, humidity, air pressure, wind, and precipitation.

**Table 3:** Description of urban air quality monitoring stations.

No.	Station	District
1142A	Fifteenth factory	Huangpu
1143A	Hongkou	Hongkou
1144A	Xuhui Shanghai Normal University	Xuhui
1145A	Yangpu Sipiao	Yangpu
1147A	Jing’an monitoring station	Jing’an
1148A	Pudong Chuansha	Pudong
1149A	Pudong monitoring station	Pudong
1150A	Pudong Zhangjiang	Pudong
